# Changing surface wax compositions and related gene expression in three cultivars of Chinese pear fruits during cold storage

**DOI:** 10.7717/peerj.14328

**Published:** 2022-11-01

**Authors:** Dan Li, Yudou Cheng, Zhonglin Shang, Junfeng Guan

**Affiliations:** 1 Institute of Biotechnology and Food Science, Hebei Academy of Agriculture and Forestry Sciences, Shijiazhuang, Hebei, China; 2 College of Life Sciences, Hebei Normal University, Shijiazhuang, Hebei, China; 3 School of Life Science and Engineering, Handan University, Handan, Hebei, China

**Keywords:** Pear cultivar, Surface wax, Cold storage, Gene expression

## Abstract

The surface wax of fruit has a significant effect on abiotic stress and fruit quality. In this study, the composition of the waxes found on fruit surfaces and the related gene expression of three different pear cultivars (Xuehua, Yali, and Yuluxiang) were investigated during cold storage. The results showed that 35 wax compositions were found on the surfaces of the three pear cultivars, mainly including C_29_ alkane, three fatty acids, two esters, three aldehydes, three fatty alcohols, and three triterpenoids. The largest amount of C_29_ alkane, three fatty acids and two esters were found in Yuluxiang (YLX) on day 90, while aldehydes with carbons of C_30_ and C_32_ were the highest in Yali (YL). Xuehua (XH) showed the largest amount of C_22_ fatty alcohol on day 180 compared to YLX and YL. Larger amounts of triterpenoids were found in XH and YL when compared to YLX. The expression levels of fifteen wax related genes (*LACS1*, *KCS2*, *KCS6*, *FDH*, *KCS20*, *GL8*, *CER10*, *CER60*, *LTPG1*, *LTP4*, *ABCG12*, *CER1L*, *CAC3*, *CAC3L*, and *DGAT1L*) reached their peak at day 45 in YLX, compared to XH and YL, their expression levels in YLX were higher to different degrees. These results suggest that the different expression patterns of wax-related genes may be closely related to the difference in wax compositions of the surface wax of three pear cultivars.

## Introduction

Surface wax protects fruit from cracking, delays the loss of nutrients and water, and prevents the invasion of pathogenic bacteria (Trivedi et al., 2019). Esters of (C_3_–C_5_) alcohol-oleic acid (or linoleic acid) and butyl ester are wax components in apples and reportedly produce a greasy-feeling peel, which is linked to a reduced sensory evaluation and market value (Zhang et al., 2019a). 1-methylcyclopene, an ethylene inhibitor, inhibited the accumulation of wax in apple and pear fruit and also retarded the fruit ripening process (Li, Cheng & Guan, 2019; Li et al., 2019). However, the removal of the surface wax in blueberries induced fruit softening, rotting, and loss of anthocyanin (Chu et al., 2018a, 2018b). In addition, the presence of surface wax produced different levels of resistance to *Alternaria* rot in pear varieties (Wu et al., 2017; Tang et al., 2017). There are few studies on the analysis in changes of wax components in different varieties of pear fruit during storage.

A series of wax related genes, such as long chain acyl-CoA synthetase (*LACS*), β-ketoacyl CoA synthetase (*KCS*), β-ketoacyl CoA reductase (*KCR*), ATP-binding cassette transporter G protein (*ABCG*) and lipid transporter protein (*LTP*), have been proved to be involved in the synthesis and transportation of wax component in fruits (Liu et al., 2015; Yang et al., 2017; Zhang et al., 2019a; Zhong et al., 2020). For example, *KCS* (*KCS2*, *KCS6*/*CER6*, *KCS9*, *KCS10/FDH1 KCS18*, *KCS20*, and *CER60*), *KCR* (*KCR1_1*, *KCR1_2*), *LACS* (*LACS1*, *LACS2*), *CER1* (an element of wax alkane synthesis), *WSD1* (wax ester synthase), *DGAT1* (diacylglycerol acyltransferase), *ABCG* (*CER5*, *WBC11*), and *LTP* (*LTPG1*, *LTP3* and *LTP4*) were involved in the wax accumulation in pears (Wu et al., 2020, 2019). In addition, *CER10* (belonging to the fatty acid elongation system), *CER2* (acyltransferase), *CER1*, and *WSD1* were found in the synthesis of aliphatic wax in grapes (Dimopoulos et al., 2020). However, there is limited research on the molecular mechanisms of different fruit peel wax characteristics caused by different genetic backgrounds during storage.

Therefore, we selected three pear varieties with distinct differences in peel characteristics (Xuehua (XH), Yali (YL), and Yuluxiang (YLX)). XH and YL pears belong to *Pyrus bretschneideri*, two ancient cultivars of Chinese pears. YLX is a recently developed pear cultivar and originates from a cross between the ‘Kuerle’ (female parent) and ‘Xuehua’ (male parent). The peel characteristics of these three pear cultivars are different and these differences are related to the wax components on the surface of fruit. The YLX pear has a smooth surface and few fruit dots, while the XH pear has a rough surface and more fruit dots, the characteristics of the YL pear falls between the XH and YLX varieties. In this study, changing wax compositions on the three pears surface and the related genes expression in the XH, YL, and YLX pears and the correlation analysis between them were investigated to determine the genes that may be involved in wax synthesis on the surface of the pears.

## Materials and Methods

### Materials

The XH, YL, and YLX pear fruits were all harvested at commercial maturity from Zhao County, Hebei Province, China on September 15, September 24, and August 18, 2015, respectively. They were transported to a laboratory within 2 h of the harvest. Fruit with uniform size and no visible mechanical defects were selected and stored at 0 °C for 270 days.

Total wax extraction was performed at day 0, 45, 90, 180, and 270 of cold storage. Two pieces of peel were sampled along the symmetrical part of the long axis of the fruit. These portions were rapidly frozen in liquid nitrogen and stored at −80 °C for RNA extraction.

### Total wax extraction

Each fruit was dipped into a succession of chloroform solutions for 30 s. Then extract solutions were mixed and added into 400 µL 0.5 g L^−1^ n-tetracosane (Accustandard Inc, New Haven, CT, USA) as the internal standard. The extracting solutions were then evaporated under a gentle flow of nitrogen gas until the samples were completely dry (Wang et al., 2014). Total wax extraction contained three replicates with five fruits for each replicate.

### Wax determination

Dried wax extracts were re-dissolved in 400 µL pyridine at 50 °C for 30 min, and then were added into 400 µL bis-N, O-trimethylsilyl trifluoroacetamide (TCI, Tokyo, Japan) for derivative reaction at 60 °C for 40 min. The mixture solution was completely dried under a slow nitrogen flow. The dried wax extract was last resolved in 2.5 mL chloroform and filtered through a 0.22 µm aperture filter before determination by gas chromatography-mass spectrometry (GC-MS) (DSQ II; Thermo Fisher Scientific, Waltham, MA, USA). A DB-1 capillary (30 m * 0.25 mm ID * 0.25 µm film thickness; Agilent Technology Inc, Palo Alto, CA, USA) was used to separate the wax components. Helium was used as the carrier gas at a rate of 1.0 mL per min. The temperature conditions were as follows: the initial temperature started at 70 °C and was increased to 200 °C at the rate of 10 °C per min and was held for 1 min; the temperature was elevated to 300 °C at the rate of 4 °C per min and was held for 20 min. The mass spectrum program settings: EI ion source (70 eV). The mass-to-charge ratio ranged from 50–650; the temperature of the inlet, ion source, and transmission was 250 °C, 300 °C, and 250 °C, respectively. A qualitative analysis of the wax components was conducted according to the NIST 09 library, and the quantitative calculations were carried out by the internal standard method (Yang et al., 2017). The wax content was expressed as µg cm^−2^.

### RNA extraction and qRT-PCR analysis of wax related genes

Total RNA extraction was conducted according to the CTAB method described by Gasic, Hernandez & Korban (2004). A single chain of cDNA was synthetized using the RT Reagent kit with gDNA Eraser (TaKaRa Biomedicals, Dalian, China). SYBR* Premix Ex Taq™ (TaKaRa Biomedicals, Dalian, China) was used for the qRT-PCR analysis of wax related genes. *Actin2* was used as the endogenous reference. The quantity analysis of the gene expression was based on the formula 2^−ΔΔCT^ (Livak & Schmittgen, 2001). The expression values of detected genes in YL at day 0 were all labeled as ‘1’ to standardize the expression of these genes in the other two varieties. Melting curves were completed at the end of the amplification reaction and they were used to test specificity of primers.

The primers of the detected genes are listed in Table S1. The primers of *LACS1*, *KCS1L*, *KCS6*, *CER10*, *ABCG11*, *ABCG12*, *CER1L*, *CAC3*, and *CAC3L* were designed by OMIGA 2.0. The primers of *ACT2* and *WSD1L* refer to Li et al. (2017) and Yang et al. (2017), respectively. The other primers refer to Wu et al. (2017).

### Statistical analysis

Three replicates were conducted for all of the assays and the values were expressed as means ± standard error. Data were analyzed by one-way ANOVA, and were compared according to Duncan (α = 0.05) and LSD (*p* < 0.05) using SPSS Statistics 22 (IBM, Armonk, NY, USA). Cluster relationships, the dimension reduction of the wax compositions, and the correlation analysis of the wax compositions and wax-related gene expression were completed using cluster heat maps, principal component analysis (PCA), and heat maps in Origin 2017 (Origin Lab, Northampton, MA, USA).

## Results

### Variations in wax composition on fruit surface in three pear cultivars during cold storage

Fruit surface waxes contained aliphatic compounds and triterpenoids in all three pear cultivars. Triterpenoids were dominant in the wax compositions of XH and YL, while aliphatic compounds were predominant in the wax of YLX (Fig. S1 and Table 1). The higher proportions of alkenes, fatty acids, and esters were found in the surface wax of YLX at a later storage stage (Fig. S1), and the contents of total alkanes, total alkenes, total fatty acids, and total esters were all highest on day 90 in YLX among the three pear cultivars (Table 1). YL showed the highest content of total aldehydes on day 90 (Table 1). The total triterpenoid content in the three cultivars all showed an upward trend before day 90; however, the amounts of triterpenoid in XH and YL were higher than in YLX during cold storage (Table 1). In addition, the content of total fatty alcohols in XH reached the peak on day 180 and was higher than YLX on day 180 (Table 1).

**Table 1:** The variation of wax composition contents (alkanes, alkenes, fatty acids, esters, aldehydes, fatty alcohols, triterpenoids, and aliphatic wax) on fruit surface in three pear varieties during cold storage.

Cultivars	Storage time (d)	Wax compositions (µg cm^−2^)							
		Alkanes	Alkenes	Fatty acids	Esters	Aldehydes	Fatty alcohols	Triterpenoids	Aliphatic wax
YLX	0	35.89 ± 5.63 **c**	2.67 ± 0.83 **e**	1.41 ± 0.18 **de**	7.68 ± 0.81 **ef**	12.03 ± 4.32 **d**	5.75 ± 0.80 **fg**	17.76 ± 2.58 **h**	65.43 ± 12.20 **gh**
	45	61.20 ± 13.66 **b**	5.02 ± 0.96 **de**	1.73 ± 0.29 **de**	27.21 ± 6.06 **c**	17.68 ± 5.13 **d**	3.29 ± 0.24 **gh**	39.64 ± 11.73 **fgh**	116.13 ± 26.14 **de**
	90	101.15 ± 5.90 **a**	10.87 ± 0.19 **a**	6.65 ± 0.82 **ab**	59.92 ± 9.73 **a**	22.23 ± 3.01 **d**	3.06 ± 0.26 **h**	93.84 ± 14.70 **e**	202.88 ± 17.72 **a**
	180	96.34 ± 14.23 **a**	9.53 ± 1.62 **abc**	8.06 ± 2.50 **a**	39.54 ± 4.97 **b**	17.75 ± 5.14 **d**	3.26 ± 0.87 **gh**	71.02 ± 4.02 **ef**	174.46 ± 17.67 **b**
	270	24.56 ± 0.54 **de**	3.24 ± 0.06 **e**	3.95 ± 0.30 **cd**	11.30 ± 0.35 **def**	10.98 ± 0.06 **d**	5.06 ± 0.19 **fgh**	9.33 ± 0.53 **h**	59.11 ± 1.50 **gh**
XH	0	7.76 ± 1.12 **g**	2.9 ± 0.86 **e**	1.29 ± 0.42 **de**	4.91 ± 1.62 **f**	17.77 ± 0.33 **d**	9.47 ± 2.37 **d**	75.51 ± 12.35 **ef**	44.11 ± 6.38 **hi**
	45	11.35 ± 1.36 **g**	3.48 ± 2.12 **e**	0.69 ± 0.12 **e**	6.02 ± 1.51 **f**	17.96 ± 10.48 **d**	4.66 ± 1.19 **fgh**	142.17 ± 11.22 **d**	44.16 ± 15.82 **hi**
	90	12.75 ± 0.95 **fg**	4.16 ± 0.64 **e**	0.97 ± 0.19 **e**	7.92 ± 2.52 **ef**	21 ± 2.03 **d**	6.31 ± 1.04 **ef**	185.69 ± 6.89 **c**	53.12 ± 6.81 **hi**
	180	16.11 ± 2.5 **efg**	3.79 ± 1.84 **e**	1.47 ± 0.46 **de**	9.75 ± 1.5 **def**	21.23 ± 9.9 **d**	15.45 ± 2.41 **a**	223.95 ± 31.78 **bc**	67.79 ± 17.05 **gh**
	270	5.54 ± 2.08 **g**	2.16 ± 1.14 **e**	0.99 ± 0.29 **e**	2.08 ± 0.16 **f**	9.16 ± 0.25 **d**	3.86 ± 0.3 **fgh**	23.91 ± 2.95 **gh**	23.79 ± 3.63 **i**
YL	0	16.48 ± 2.69 **efg**	7.64 ± 2.23 **bc**	2.79 ± 2.04 **cde**	18.28 ± 5.28 **d**	51.2 ± 10.5 **b**	12.98 ± 1.88 **bc**	199.39 ± 49.18 **c**	109.36 ± 20.74 **ef**
	45	14.85 ± 2.23 **efg**	6.83 ± 2.22 **cd**	2.04 ± 1.14 **de**	16.82 ± 1.77 **de**	38.43 ± 13.78 **c**	5.58 ± 0.43 **fgh**	224.24 ± 39.53 **bc**	84.54 ± 20.42 **fg**
	90	22.87 ± 2.63 **def**	10.32 ± 2.09 **ab**	5.14 ± 3.31 **bc**	35.1 ± 10.66 **bc**	63.68 ± 2.29 **a**	12.11 ± 2.53 **c**	261.69 ± 30.35 **b**	149.21 ± 20.51 **bc**
	180	30.67 ± 1.75 **cd**	8.16 ± 2.12 **bc**	1.64 ± 2.6 **de**	39 ± 9.92 **b**	44.34 ± 12.2 **bc**	14.73 ± 1.7 **ab**	300.39 ± 23.44 **a**	138.54 ± 23.82 **cd**
	270	13.31 ± 2.04 **fg**	2.45 ± 0.28 **e**	1.45 ± 0.3 **de**	7.19 ± 0.5 **ef**	22.44 ± 2.41 **d**	8.22 ± 0.63 **de**	58.1 ± 1.79 **efg**	55.06 ± 5.16 **gh**

**Note:**

Data were mean ± SE (*n* = 3); data in the same column labeled with different letters represent significant differences of *p* < 0.05 according to one-way ANOVA. The bold letters represent significant differences in the data of the same column.

**Figure 1:**
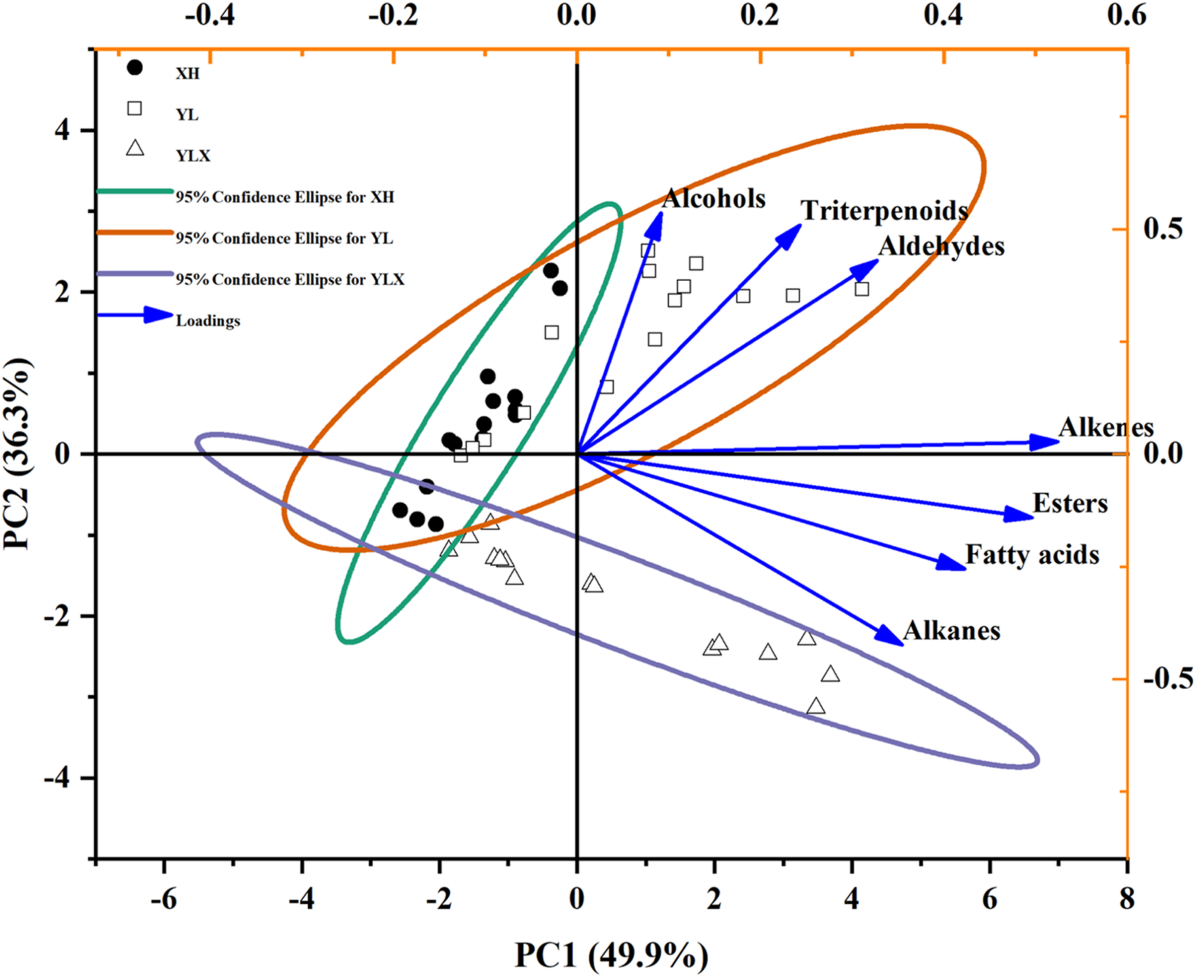
PCA of seven wax compositions on fruit surface in three pear varieties during cold storage.

In order to further identify the differences in waxes among the three varieties during storage, PCA results showed that the cumulative variance contribution rate of PC1 (49.9%) and PC2 (36.3%) was 86.2%. This could represent the seven wax components used to evaluate the effects of variety and storage time on the surface wax of three pear cultivars (Fig. 1). Fatty alcohols, triterpenoids, and aldehydes possessed the load on the positive axis of PC1 and PC2, however, alkenes, esters, fatty acids, and alkanes occupied the load on the positive axis of PC1 and the negative axis of PC2 (Fig. 1). The three pear cultivars were divided into two groups at a 95% confidence: wax composition, such as alkenes, esters, fatty acids, and alkanes reflected the surface wax characteristics of YLX, while fatty alcohols, triterpenoids, and aldehydes represented the surface wax features of XH and YL (Fig. 1).

### Surface wax profiles and variations among three pear cultivars during cold storage

**Figure 2:**
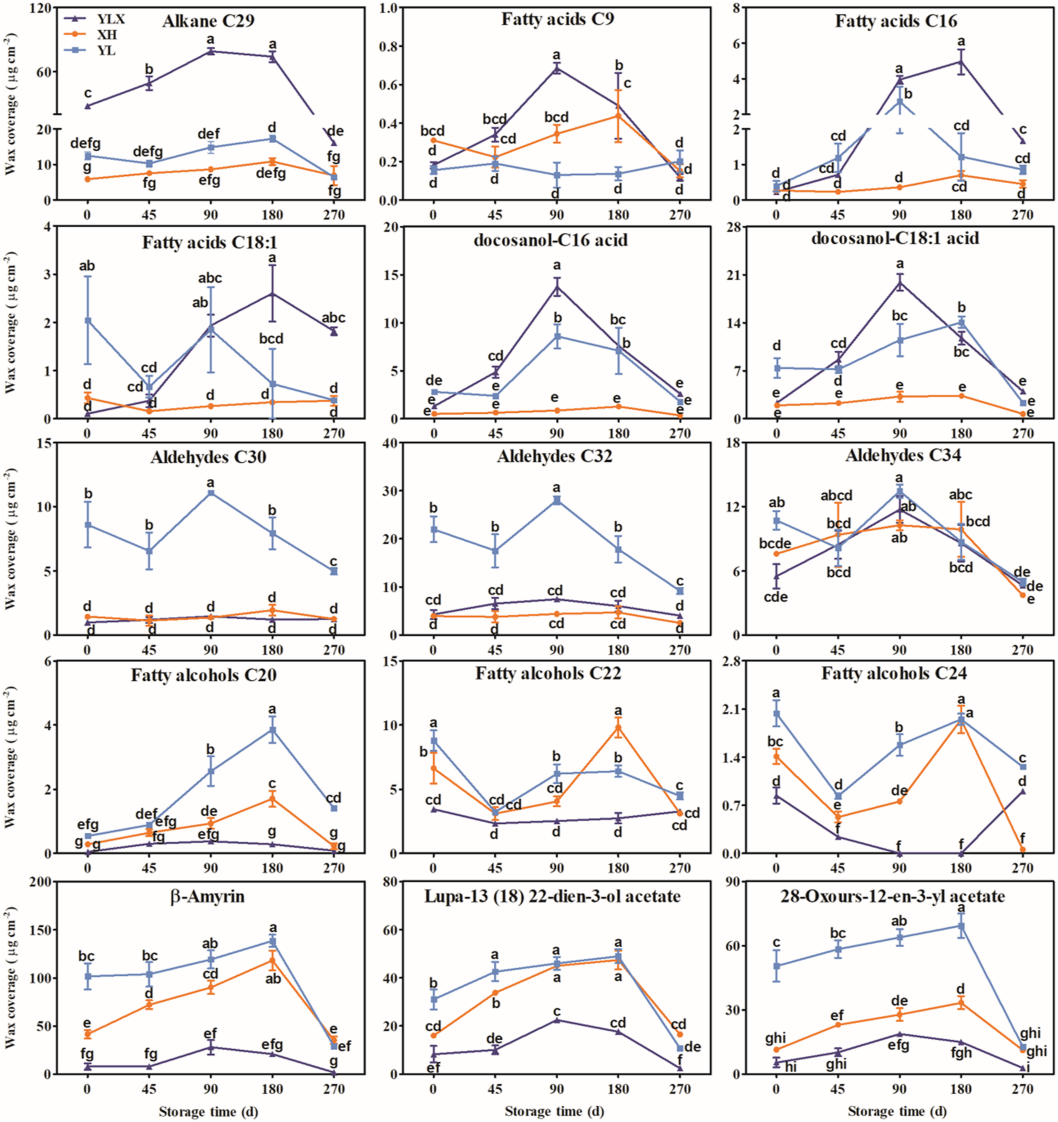
The contents changes of main wax compositions on fruit surface in three pear varieties during cold storage. Data were mean ± SE (*n* = 3); data labeled with different letters represent significant differences of *p* < 0.05 according to one-way ANOVA.

Alkanes with C_21_–C_31_ chains, three fatty acids, two esters, aldehydes with C_24_–C_34_ chains, fatty alcohols with C_16_–C_28_ chains, and seven triterpenoids were found in the three pear cultivars (Figs. S2–S7). The waxes were mainly composed of C_29_ alkane, three fatty acids, two esters, three aldehydes (C_30_, C_32_ and C_34_), three fatty alcohols (C_20_, C_22_ and C_24_), and three triterpenoids (Fig. 2 and Figs. S2–S7). The contents of C_29_ alkane, the three fatty acids, and two esters all peaked on day 90 and were highest in YLX compared with the other two varieties (Fig. 2). The YL cultivar recorded the two highest aldehyde contents of C_30_ and C_32_ before day 90 and on day 90 of cold storage (Fig. 2). The content of the three fatty alcohols (C_20_, C_22_ and C_24_) in XH all showed an upward trend before day 90, and peaked on day 90 (Fig. 2). In addition, the contents of the three main triterpenoids all reached peak values on day 90 in all three pear cultivars. They were higher in XH and YL than in YLX (Fig. 2).

Thirty-five wax compositions were clustered into two categories (Fig. S8). Each component of the alkanes, fatty acids, and esters all belonged to one category, while each compound of aldehydes (except for the composition C_33_), fatty alcohols, and triterpenoids (except for [(3β,5α)-4,4-dimethylcholesta-8,24-dien-3-yl] oxy) were classified into another category (Fig. S8).

### Expression pattern of wax related genes in three pear cultivars during cold storage

**Figure 3:**
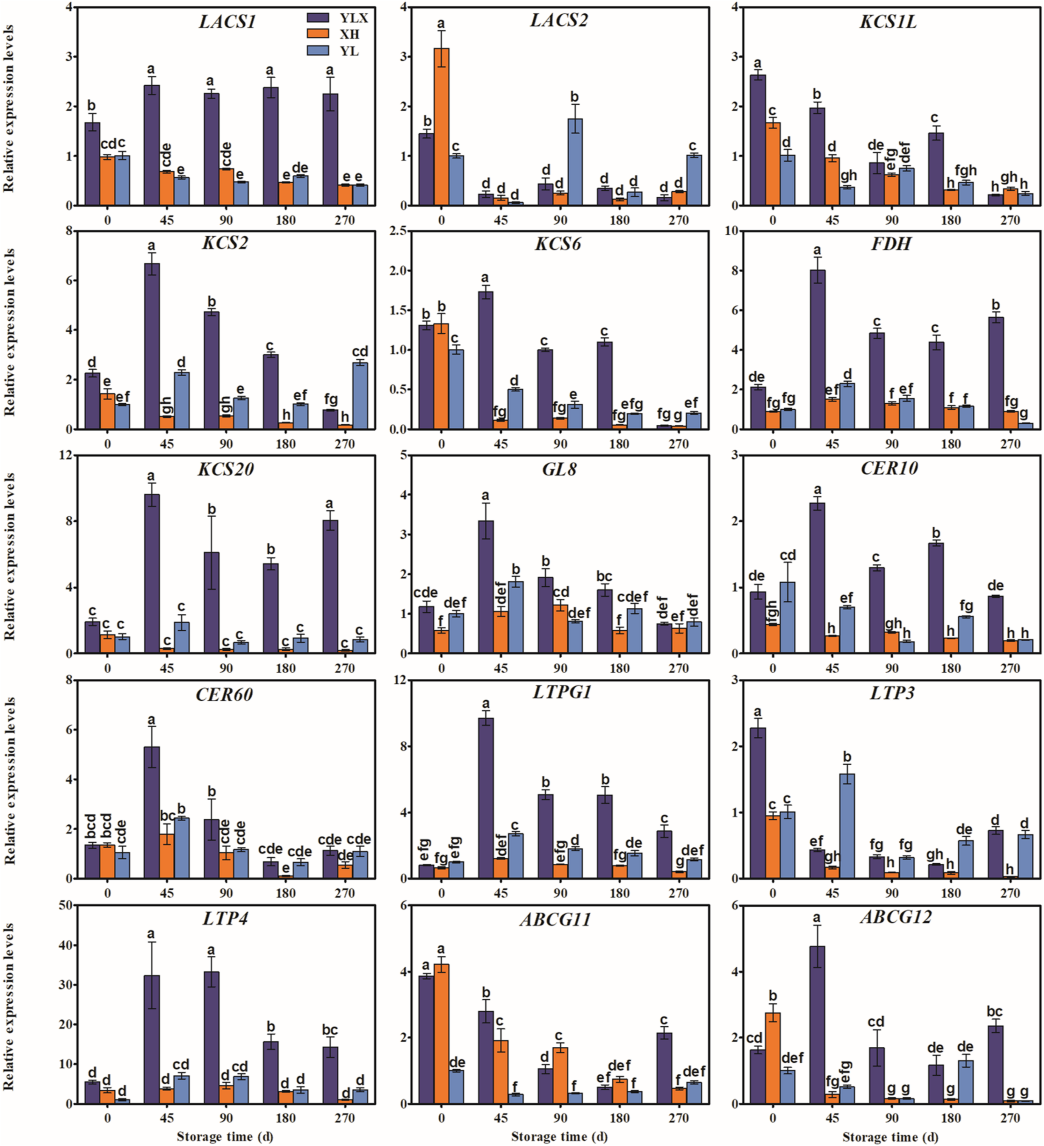
Expression pattern of wax biosynthesis and transporter protein genes of peel in three pear cultivars during cold storage. Data were mean ± SE (*n* = 3); data labeled with different letters represent significant differences of *p* < 0.05 according to one-way ANOVA.

**Figure 4:**
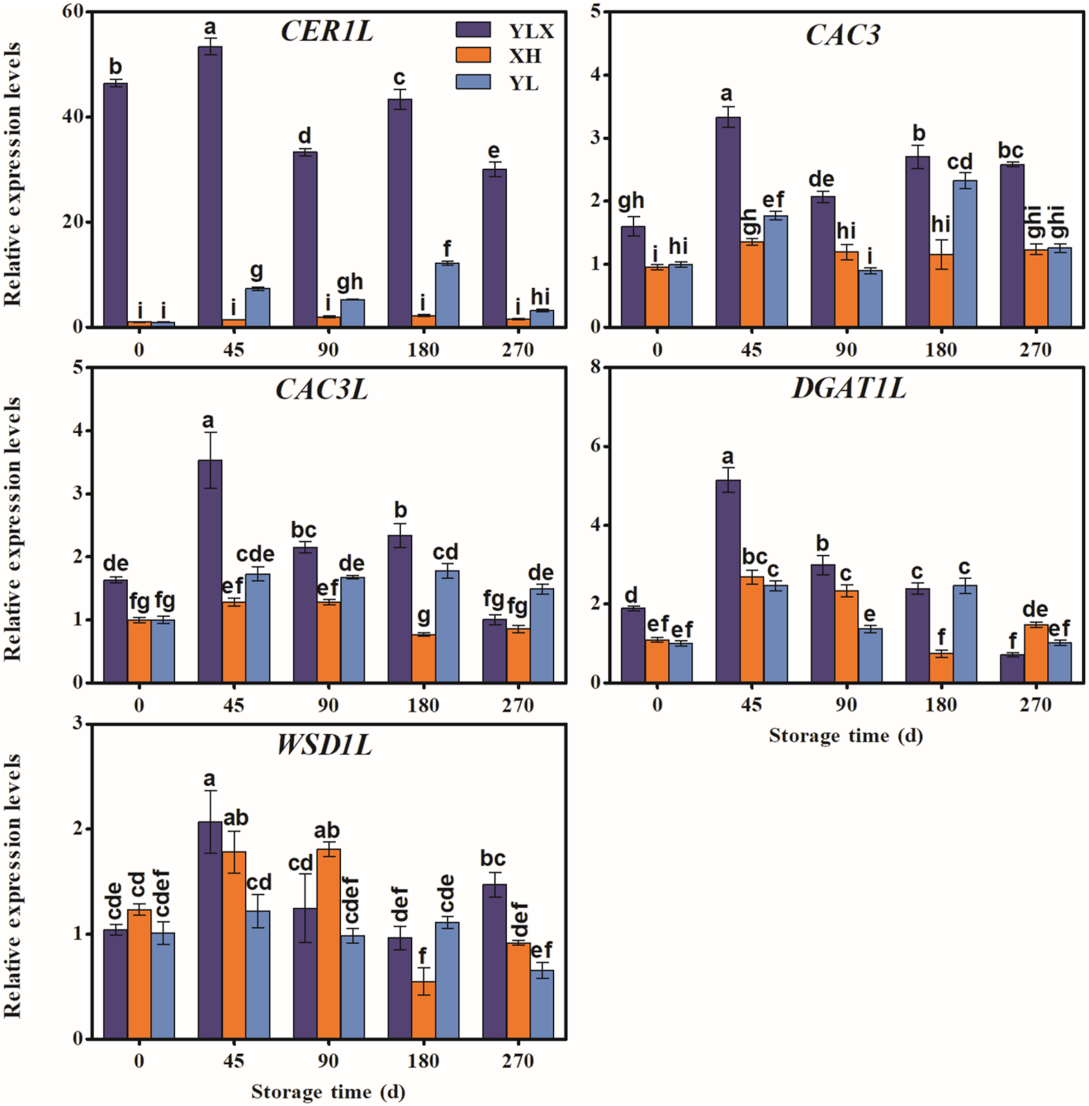
Expression pattern of biosynthesis genes of some wax composition groups of peel in three pear cultivars during cold storage. Data were mean ± SE (*n* = 3); data labeled with different letters represent significant differences of *p* < 0.05 according to one-way ANOVA.

The expression levels of the fifteen genes (*LACS1*, *KCS2*, *KCS6*, *FDH*, *KCS20*, *GL8*, *CER10*, *CER60*, *LTPG1*, *LTP4*, *ABCG12*, *CER1L*, *CAC3*, *CAC3L*, and *DGAT1L*) in the peel of YLX all peaked at day 45, and were higher than those in XH and YL (Figs. 3 and 4). Figures 3 and 4 showed the increasing trend of the relative expression abundance of 10 genes (*KCS2*, *FDH*, *GL8*, *CER60*, *LTPG1*, *LTP3*, *CER1L*, *CAC3*, *CAC3L*, *DGAT1L*) in the peel of YL prior to day 45. In addition, the expression levels of the transcripts of nine genes (*LACS1*, *FDH*, *KCS20*, *CER10*, *CER60*, *LTPG1*, *LTP4*, *CER1L*, *CAC3L*) in the peel of XH were unchanged; however, only the expression abundances of another three genes (*GL8*, *CAC3*, and *DGAT1L*) in XH increased before day 45. The expression levels of *WSD1L* all showed a rising trend before day 45, but no significant differences were found at day 45 among three pear cultivars (Fig. 4).

### Correlation analysis between wax composition content and related gene expression levels in three pear cultivars

**Figure 5:**
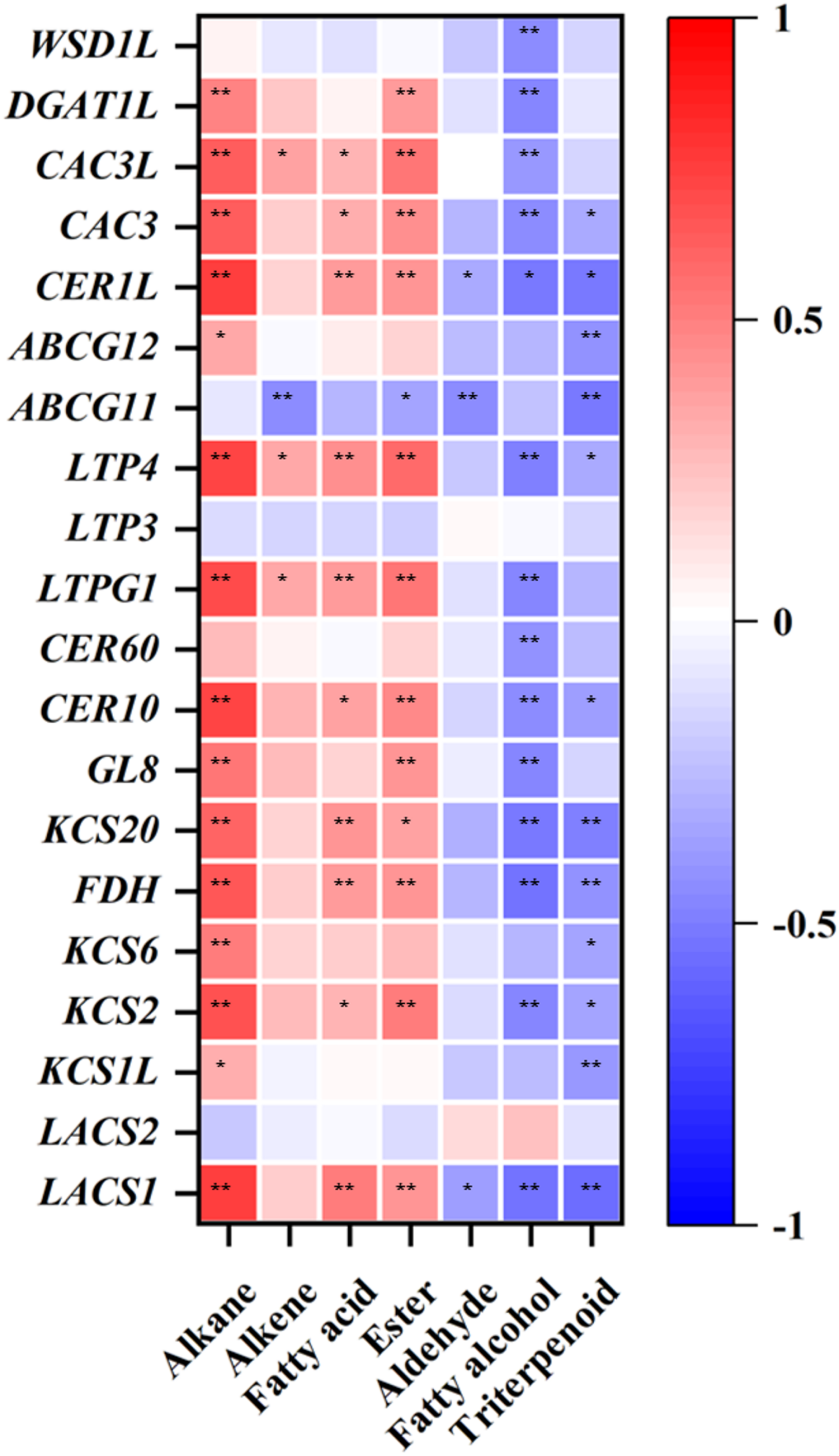
Correlation coefficient analysis between the wax-related gene expression levels and the corresponding wax composition contents in three pear varieties during cold storage. One asterisk (*) and two asterisks (**) represent a correlation at *p* < 0.05 and *p* < 0.01, respectively.

A positive correlation existed between the mRNA expression of three genes (*LTPG1*, *LTP4*, and *CAC3L*) and the content of alkanes, alkenes, fatty acids and esters in the waxes (*p* < 0.05 or *p* < 0.01) (Fig. 5). The expression levels of *LACS1*, *KCS2*, *FDH*, *KCS20*, *CER10*, *CER1L*, and *CAC3* showed a positive correlation with alkane, fatty acid, and ester content at *p* < 0.05 or *p* < 0.01(Fig. 5). The expression levels of *KCS1L*, *KCS6*, and *ABCG12* were only positively related to the content of alkanes, and the expression patterns of *GL8* and *DGAT1L* showed a positive correlation with the contents of alkanes and esters at *p* < 0.05 or *p* < 0.01, respectively (Fig. 5). In addition, the transcription levels of the 20 detected genes, except for *LACS2*, were negatively correlated with the contents of aldehydes, fatty alcohols, and triterpenoids in varying degrees (Fig. 5).

## Discussion

Fruit waxes typically contained aliphatic wax and triterpenoids, of which C_29_ alkane and triterpenoids made up a high proportion in apple and blueberry fruits (Chu et al., 2017; Curry, 2008). In this study, we also found that alkanes with odd-chains (primarily composed of C_29_ alkane), three aliphatic compositions (fatty acids, fatty alcohols, and aldehydes) with even-chains, and three triterpenoids including β-amyrin, were predominant in the wax of the three pear cultivars (Figs. S2–S7). Similar fruit wax profiles were also found in the Pingguoli pear and navel orange (Wang et al., 2014; Yin et al., 2011).

Differences in fruit wax compositions were related to fruit peel characteristics and their genetic backgrounds. The XH and YL types belonged to the white pear (*Pyrus bretschneideri*) and exhibited a rough surface and obvious fruit dots, while YLX (a hybrid of XH and Kuerle) was similar in appearance to the Kuerle fragrant pear (*Pyrus sinkiangensis*) with a smooth peel and small fruit dots. The Hongxiangsu pear (a hybrid of Eli and Kuerle), as the sister strain of YLX, had similar peel characteristics as YLX, and also had a higher content of aliphatic wax than triterpenoids (Li, Cheng & Guan, 2019). Thus, it was inferred that the surface wax properties of the YLX and Hongxiangsu pears may be inherited by the Kuerle pear. It has been reported that the cuticular wax of YLX was phylogenetically similar to the surface wax genetic characteristics of Kuerle. The results also showed that YLX and Hongxiangsu had similar wax crystal morphologies (Wu et al., 2018). Similar results were obtained in our previous study, where the Hongxiangsu pear had a similar fruit wax composition and proportion with YLX (Li, Cheng & Guan, 2019). Thus, the smooth peel of YLX may be caused by the higher ratio of aliphatic wax on the fruit’s surface. XH acted as the male parent of YLX and had completely different wax characteristics than YLX (Fig. 2, Fig. S8, and Table 1). Compared to YLX, XH contained lesser amounts of aliphatic wax and higher proportions of triterpenoids on the fruit surface, resulting in its characteristics of rough surface.

Previous investigation found that fruit surface wax contained higher proportions of alkane and lower triterpenoid content, this wax profile reduced water loss in pepper (Parsons et al., 2012). The wax profiles of grape and blueberry were similar, in which triterpenoids were the main component (Chu et al., 2018a; Pensec et al., 2014). The accumulation of the C_29_ and C_31_ alkanes on the surface of leaves promoted drought tolerance in watermelon (Li et al., 2020). In this study, YLX exhibited different wax characteristics than the other two pear varieties and had a higher proportion and content of C_29_ alkane, but a lower proportion of triterpenoids in its surface wax (Fig. 2 and Table 1).

*MdCER1*, *MdCER2*, and *MdKCS1* are the three genes responsible for wax synthesis and transportation, all promoted the formation of fruit wax in response to abiotic stress in apple (Qi et al., 2019; Zhang et al., 2019b; Zhong et al., 2020). *KCS9*, *KCS20*, *FDH*, *CER60*, *LTP4*, and *LTPG1* were related to the accumulation of total wax contents in pear fruit (Wu et al., 2020). *CER10*, *WSD1*, *CER1*, *CsKCS2*, and *CsKCS11* were involved in the synthesis of the aliphatic wax of grape and citrus (Dimopoulos et al., 2020; Yang et al., 2021). In this research, we found the maximum expression levels of *LACS1*, *KCS2*, *KCS6*, *FDH*, *KCS20*, *GL8* (encoding KCR enzyme), *CER10*, *CER60*, *LTPG1*, *LTP4*, and *ABCG12* in YLX by combining correlation analysis of wax components and wax related genes (Fig. 5). These results were observed before the total contents of alkanes, fatty acids, and esters peaked (Fig. 3). A positive correlation also existed between the expression amounts of the above genes and their corresponding compositions, with the exception of *CER60* (Fig. 5). It was speculated that more than 10 genes may be involved in the synthesis of aliphatic wax in pear.

**Figure 6:**
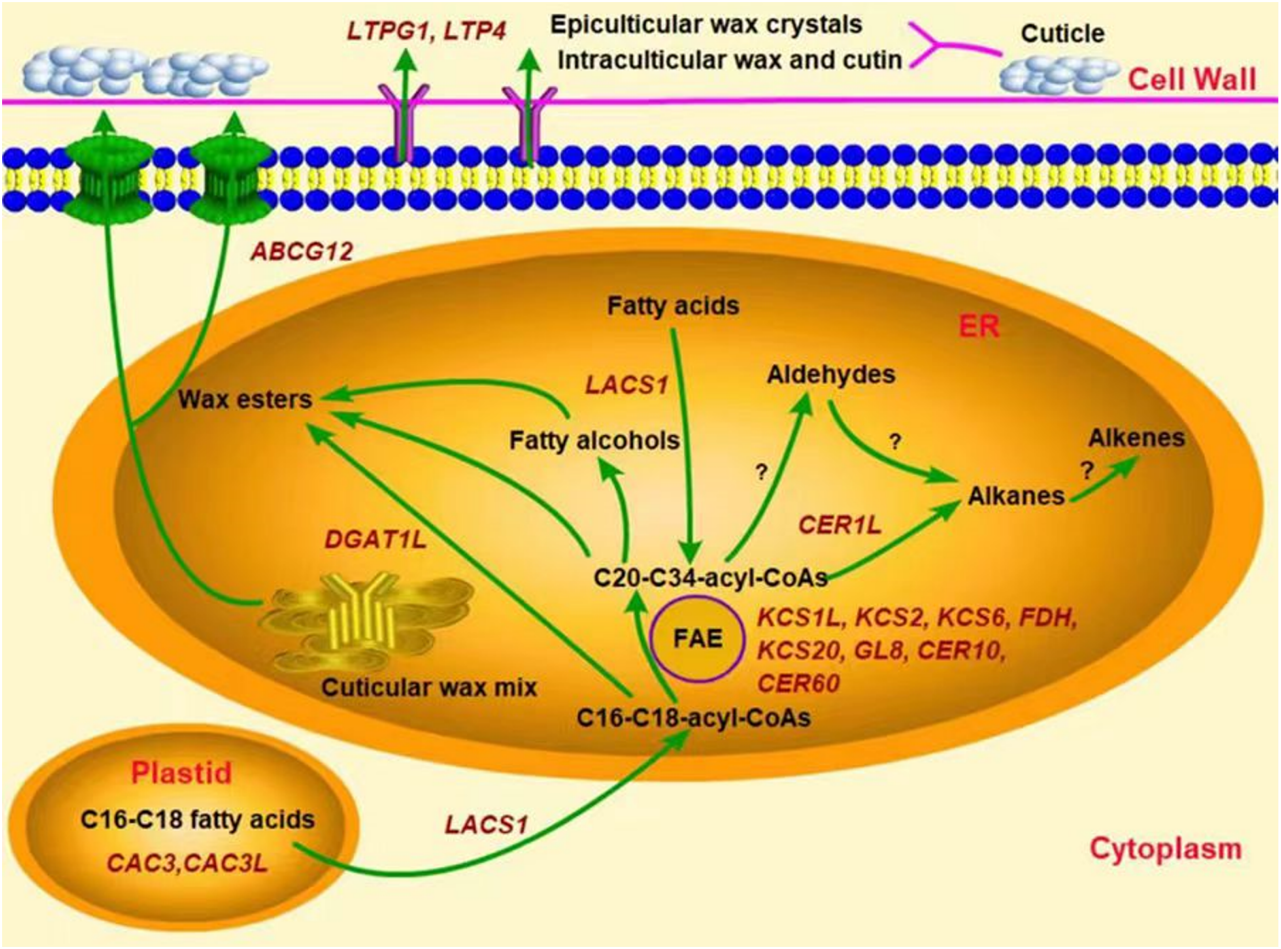
Predicted model of the wax related genes involved in wax synthesis and transport in pear fruit.

In addition, several genes were associated with the synthesis of some wax components, such as the alkane synthesis related genes *CER1L*, *CER1-1*, and *CER1-3* (Pascal et al., 2019; Yang et al., 2022), the C_16_ fatty acid synthesis genes *CAC3* and *CAC3L*, and the wax ester synthesis gene *WSD1* (Li et al., 2008; Lü et al., 2011). Our results showed that the peak transcriptional levels of *CER1L*, *CAC3* and *CAC3L*, and *DGAT1L* (also acting as wax ester synthesis gene) appeared before the maximum accumulation of their corresponding wax composition (alkanes, fatty acids and wax esters) in the YLX pear (Fig. 4 and Table 1). The expression levels of the first three genes also showed a close positive correlation with the biosynthesis of three compositions of alkanes, fatty acids, and esters in the fruit wax of three pear cultivars (Fig. 5). In contrast, the aliphatic wax content on the fruit surface hardly increased in XH during the entire storage period, and no distinct variations were found in the expression of wax-related genes in the peel of XH. The YL variety showed less expression in the amounts of wax-related genes, including *KCS2, FDH, GL8 (KCR), CER60, LTPG1, CER1L, CAC3, CAC3L*, and *DGAT1L* (Figs. 3 and 4), when compared to YLX. Their transcription levels were significantly increased at day 45, which was consistent with the amount of aliphatic wax accumulation (Figs. 3–5). The total contents of alkanes, fatty acids, and esters, as well as C_16_ fatty acid and two main esters all trended upward in YL; however, they were fewer in amount compared to YLX (Fig. 2 and Table 1). Therefore, wax related genes, such as *LACS1*, *KCS2*, *KCS6*, *FDH*, *KCS20*, *GL8*, *CER10*, *LTPG1*, *LTP4*, *ABCG12*, *CER1L*, *CAC3*, *CAC3L*, and *DGAT1L*, may be involved in fruit wax accumulation in three pear cultivars during cold storage. We established a model for the wax synthesis pathway in pear, which lays a foundation for the functional study of the genes involved in wax synthesis of the surface in different pear varieties (Fig. 6).

## Conclusions

In this study, the PCA analysis of fruit wax compositions showed that YLX belonged to one composition group, while XH and YL were part of another group. The differences of fruit wax compositions in the three cultivars may be related to different wax-related gene expressions, in which there was a higher expression of the fifteen wax-related genes in YLX *vs* in XH and YL at day 45 of cold storage.

## Supplemental Information

10.7717/peerj.14328/supp-1 Supplemental Information 1 The proportion variation of wax composition (alkanes, alkenes, fatty acids, esters, aldehydes, fatty alcohols and triterpenoids) on fruit surface in three pear cultivars during cold storage. (A) day 0 (B) day 45 (C) day 90 (D) day 180 (E) day 270. Yuluxiang, Xuehua and Yali are abbreviated to YLX, XH and YL.

10.7717/peerj.14328/supp-2 Supplemental Information 2 Profile of carbon chains length of wax alkanes on fruit surface in three pear cultivars during cold storage. Data were mean ± SE (*n* = 3); different letters marked on the three columns of the same group indicate significant differences in data according to one-way ANOVA at *p* < 0.05. (A) day 0 (B) day 45 (C) day 90 (D) day 180 (E) day 270. Yuluxiang, Xuehua and Yali are abbreviated to YLX, XH and YL.

10.7717/peerj.14328/supp-3 Supplemental Information 3 Profile of carbon chains length of wax fatty acids on fruit surface in three pear cultivars during cold storage. Data were mean ± SE (*n* = 3); different letters marked on the three columns of the same group indicate significant differences in data according to one-way ANOVA at *p* < 0.05. (A) day 0 (B) day 45 (C) day 90 (D) day 180 (E) day 270. Yuluxiang, Xuehua and Yali are abbreviated to YLX, XH and YL.

10.7717/peerj.14328/supp-4 Supplemental Information 4 Profile of carbon chains length of wax esters on fruit surface in three pear cultivars during cold storage. Data were mean ± SE (*n* = 3); different letters marked on the three columns of the same group indicate significant differences in data according to one-way ANOVA at *p* < 0.05. (A) day 0 (B) day 45 (C) day 90 (D) day 180 (E) day 270. Yuluxiang, Xuehua and Yali are abbreviated to YLX, XH and YL.

10.7717/peerj.14328/supp-5 Supplemental Information 5 Profile of carbon chains length of wax aldehydes on fruit surface in three pear cultivars during cold storage. Data were mean ± SE (*n* = 3); different letters marked on the three columns of the same group indicate significant differences in data according to one-way ANOVA at *p* < 0.05. (A) day 0 (B) day 45 (C) day 90 (D) day 180 (E) day 270. Yuluxiang, Xuehua and Yali are abbreviated to YLX, XH and YL.

10.7717/peerj.14328/supp-6 Supplemental Information 6 Profile of carbon chains length of wax fatty alcohols on fruit surface in three pear cultivars during cold storage. Data were mean ± SE (*n* = 3); different letters marked on the three columns of the same group indicate significant differences in data according to one-way ANOVA at *p* < 0.05. (A) day 0 (B) day 45 (C) day 90 (D) day 180 (E) day 270. Yuluxiang, Xuehua and Yali are abbreviated to YLX, XH and YL.

10.7717/peerj.14328/supp-7 Supplemental Information 7 Profile of wax triterpenoids on fruit surface in three pear cultivars during cold storage. Data were mean ± SE (*n* = 3); different letters marked on the three columns of the same group indicate significant differences in data according to one-way ANOVA at *p* < 0.05. (A) day 0 (B) day 45 (C) day 90 (D) day 180 (E) day 270. Yuluxiang, Xuehua and Yali are abbreviated to YLX, XH and YL.

10.7717/peerj.14328/supp-8 Supplemental Information 8 Cluster heatmap of thirty-five wax compositions on fruit surface in three pear varieties. Color indicated wax composition contents, and each column represented a combination of variety and storage period. Yellow square areas represent varieties and time combinations with higher wax content after clustering. The wax component indicated by blue asterisk represents the dividing point of component clustering.

10.7717/peerj.14328/supp-9 Supplemental Information 9 Primers of determined genes.

10.7717/peerj.14328/supp-10 Supplemental Information 10 Raw data. The identification results of wax components of three pears stored at low temperature, the changes of content and the quantitative expression of wax related synthesis and transport genes in this process.

## Additional Information and Declarations

### Data Availability

The following information was supplied regarding data availability: The raw measurements are available in the Supplemental Files.
