# Supplementary figures and images for: Changing surface wax compositions and related gene expression in three cultivars of Chinese pear fruits during cold storage
Source: PeerJ. 2022 Nov 1;10:e14328. doi: 10.7717/peerj.14328 (PMC9635359; doi:10.7717/peerj.14328)

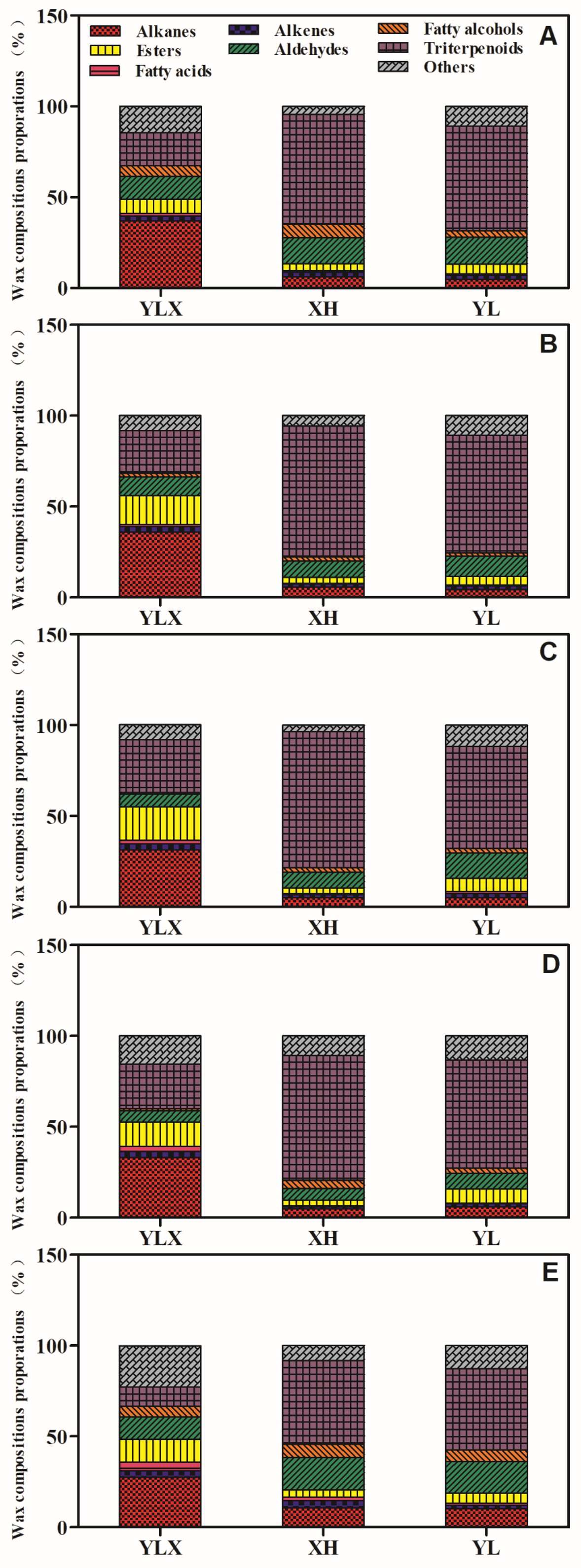

Supplement: Supplemental Information 1 — (A) day 0 (B) day 45 (C) day 90 (D) day 180 (E) day 270. Yuluxiang, Xuehua and Yali are abbreviated to YLX, XH and YL. [file peerj-10-14328-s001.png]

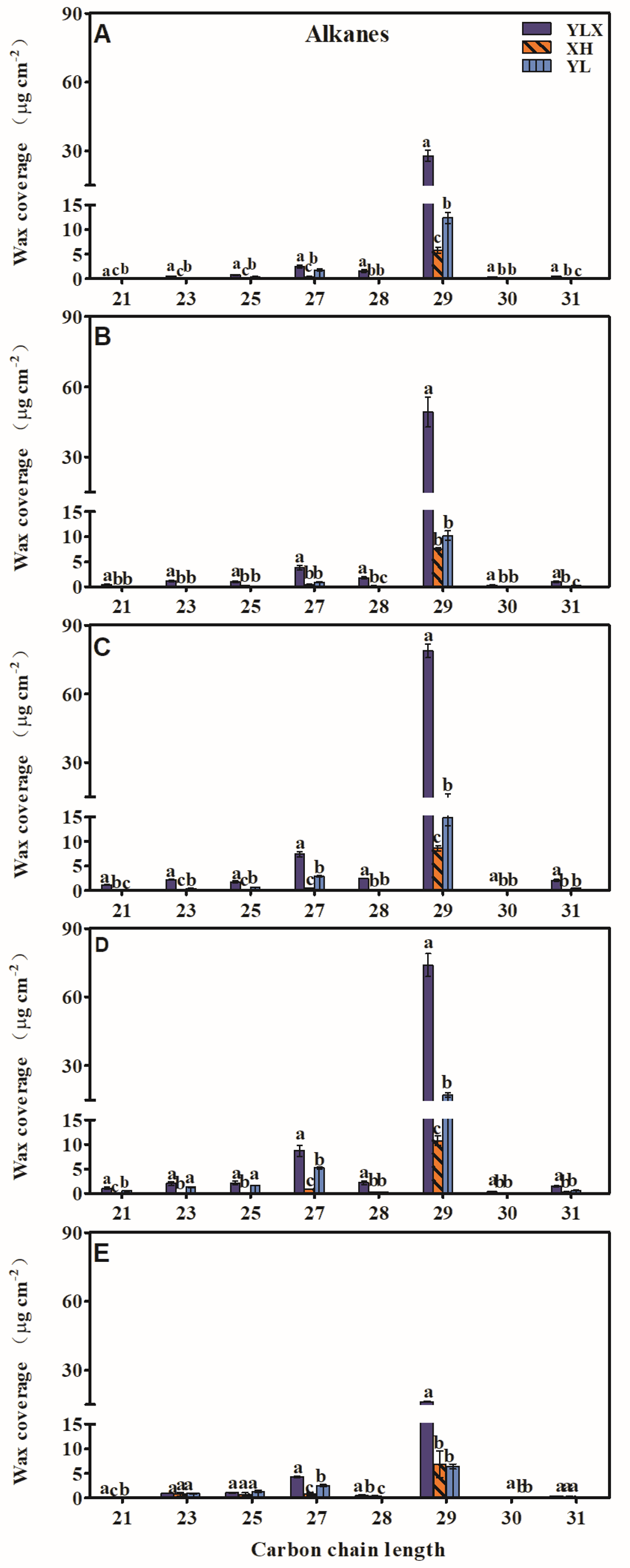

Supplement: Supplemental Information 2 — Data were mean ± SE (n = 3); different letters marked on the three columns of the same group indicate significant differences in data according to one-way ANOVA at p < 0.05. (A) day 0 (B) day 45 (C) day 90 (D) day 180 (E) day 270. Yuluxiang, Xuehua and Yali are abbreviated to YLX, XH and YL. [file peerj-10-14328-s002.png]

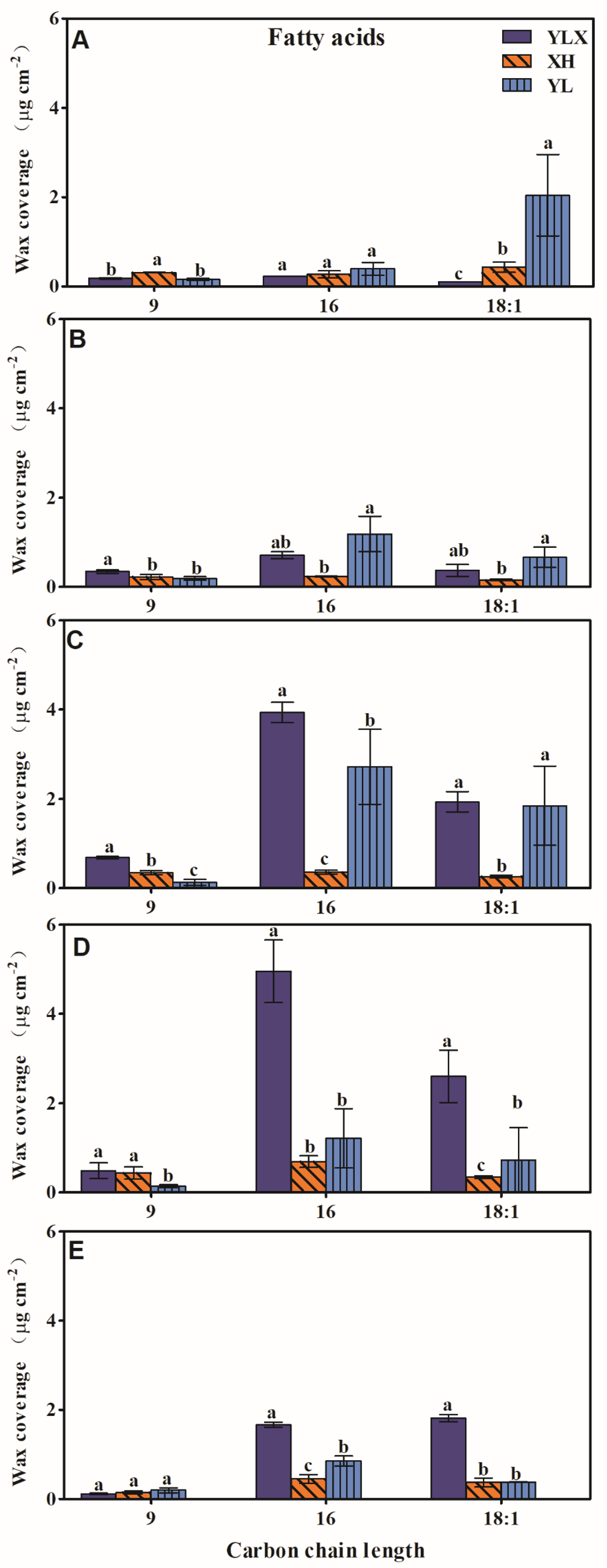

Supplement: Supplemental Information 3 — Data were mean ± SE (n = 3); different letters marked on the three columns of the same group indicate significant differences in data according to one-way ANOVA at p < 0.05. (A) day 0 (B) day 45 (C) day 90 (D) day 180 (E) day 270. Yuluxiang, Xuehua and Yali are abbreviated to YLX, XH and YL. [file peerj-10-14328-s003.png]

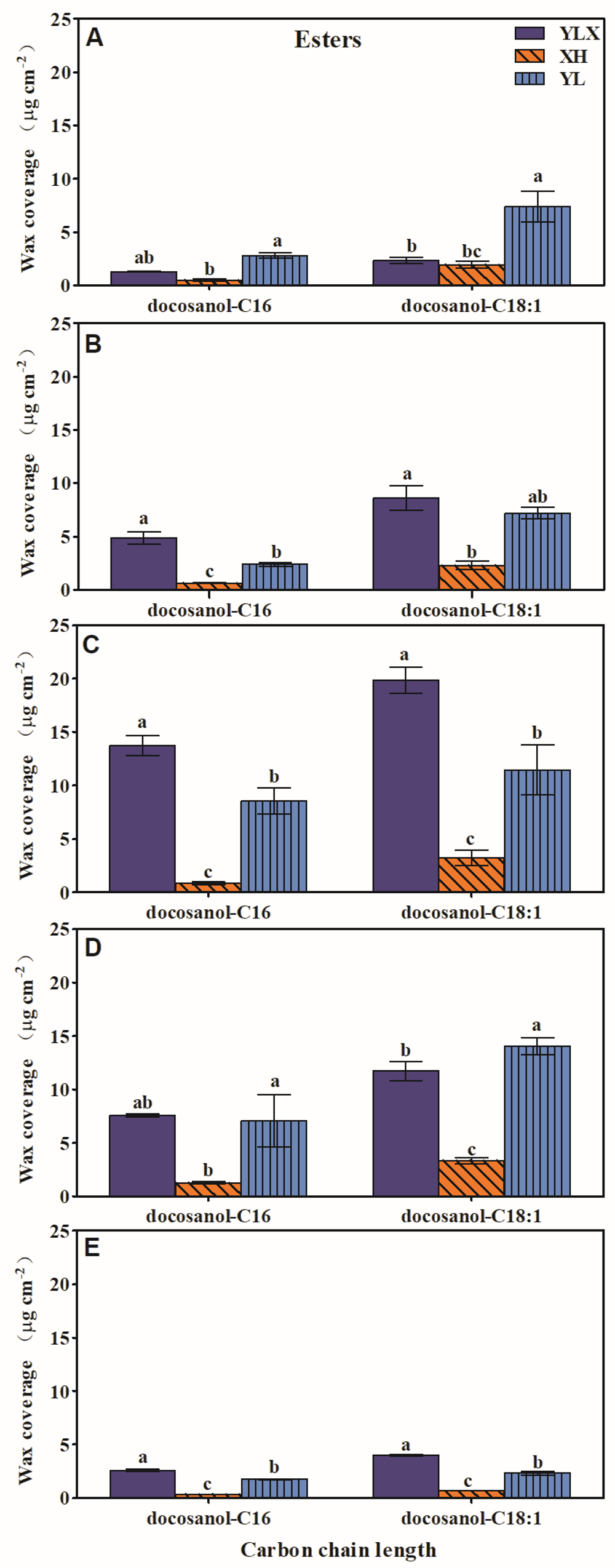

Supplement: Supplemental Information 4 — Data were mean ± SE (n = 3); different letters marked on the three columns of the same group indicate significant differences in data according to one-way ANOVA at p < 0.05. (A) day 0 (B) day 45 (C) day 90 (D) day 180 (E) day 270. Yuluxiang, Xuehua and Yali are abbreviated to YLX, XH and YL. [file peerj-10-14328-s004.png]

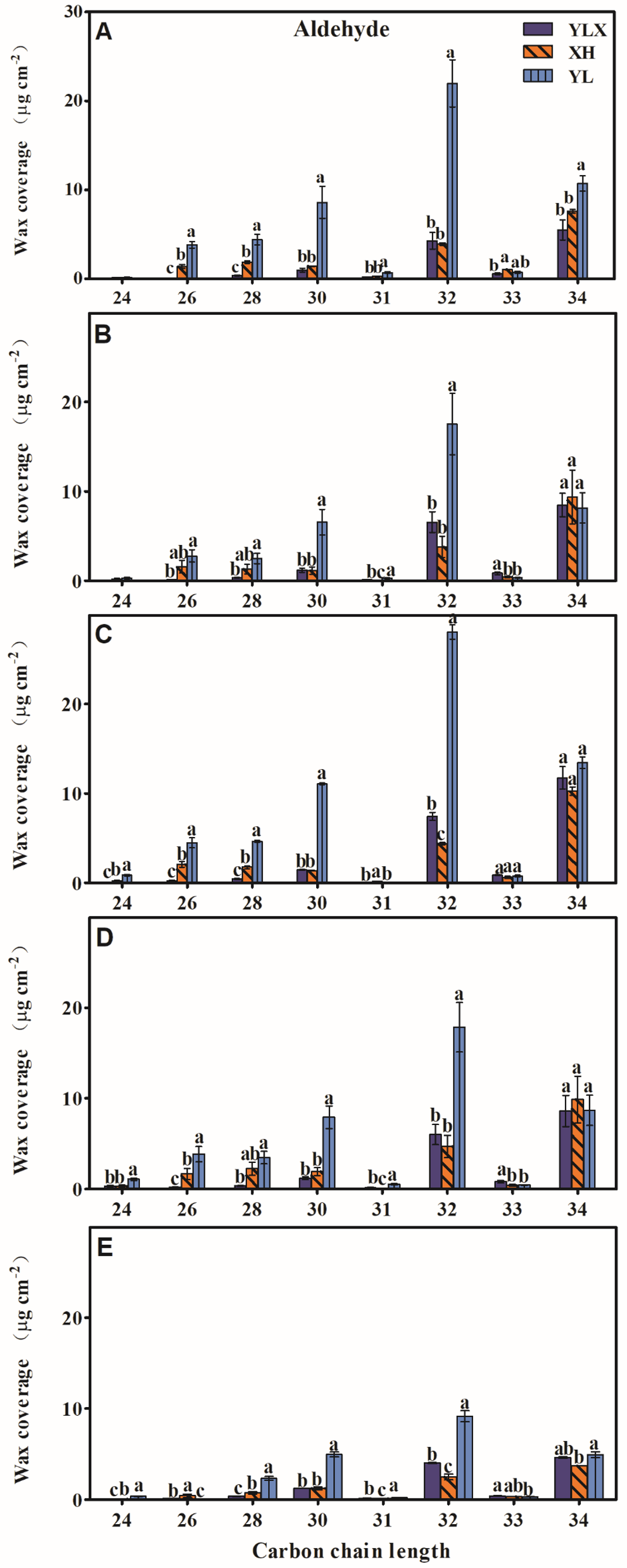

Supplement: Supplemental Information 5 — Data were mean ± SE (n = 3); different letters marked on the three columns of the same group indicate significant differences in data according to one-way ANOVA at p < 0.05. (A) day 0 (B) day 45 (C) day 90 (D) day 180 (E) day 270. Yuluxiang, Xuehua and Yali are abbreviated to YLX, XH and YL. [file peerj-10-14328-s005.png]

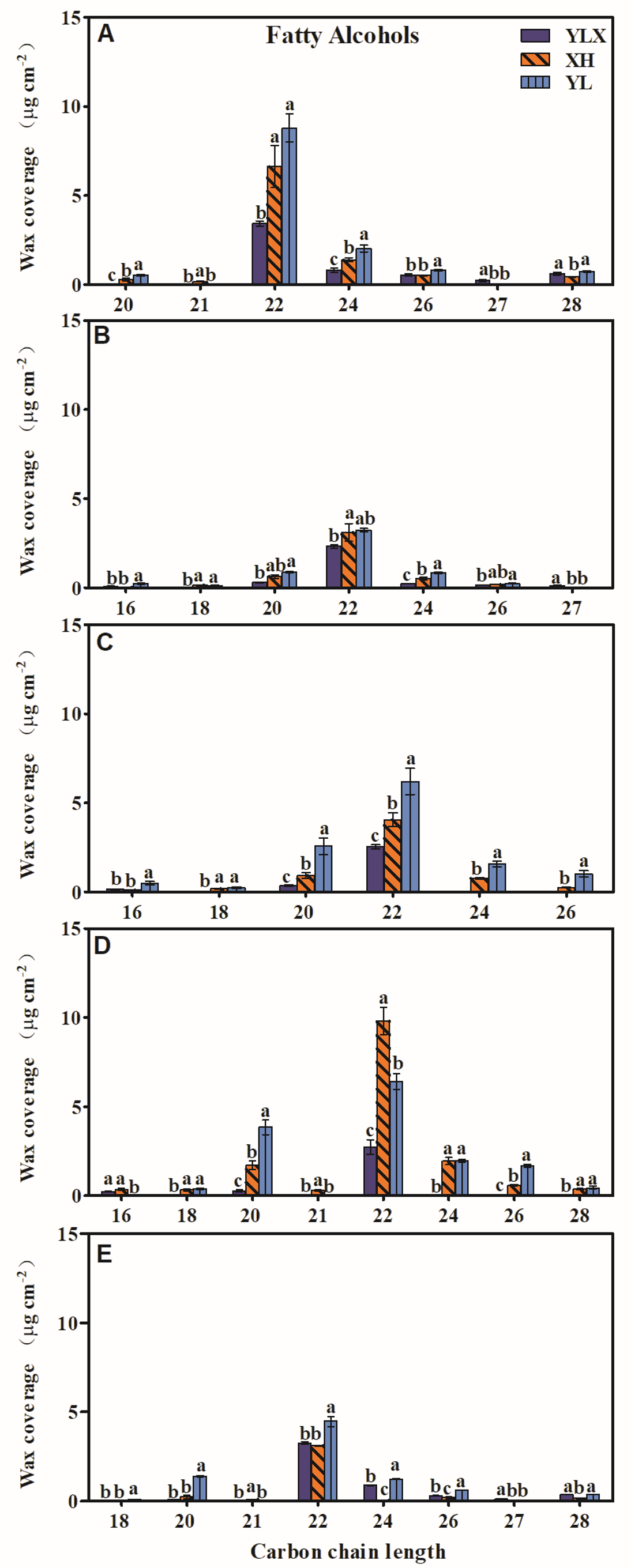

Supplement: Supplemental Information 6 — Data were mean ± SE (n = 3); different letters marked on the three columns of the same group indicate significant differences in data according to one-way ANOVA at p < 0.05. (A) day 0 (B) day 45 (C) day 90 (D) day 180 (E) day 270. Yuluxiang, Xuehua and Yali are abbreviated to YLX, XH and YL. [file peerj-10-14328-s006.png]

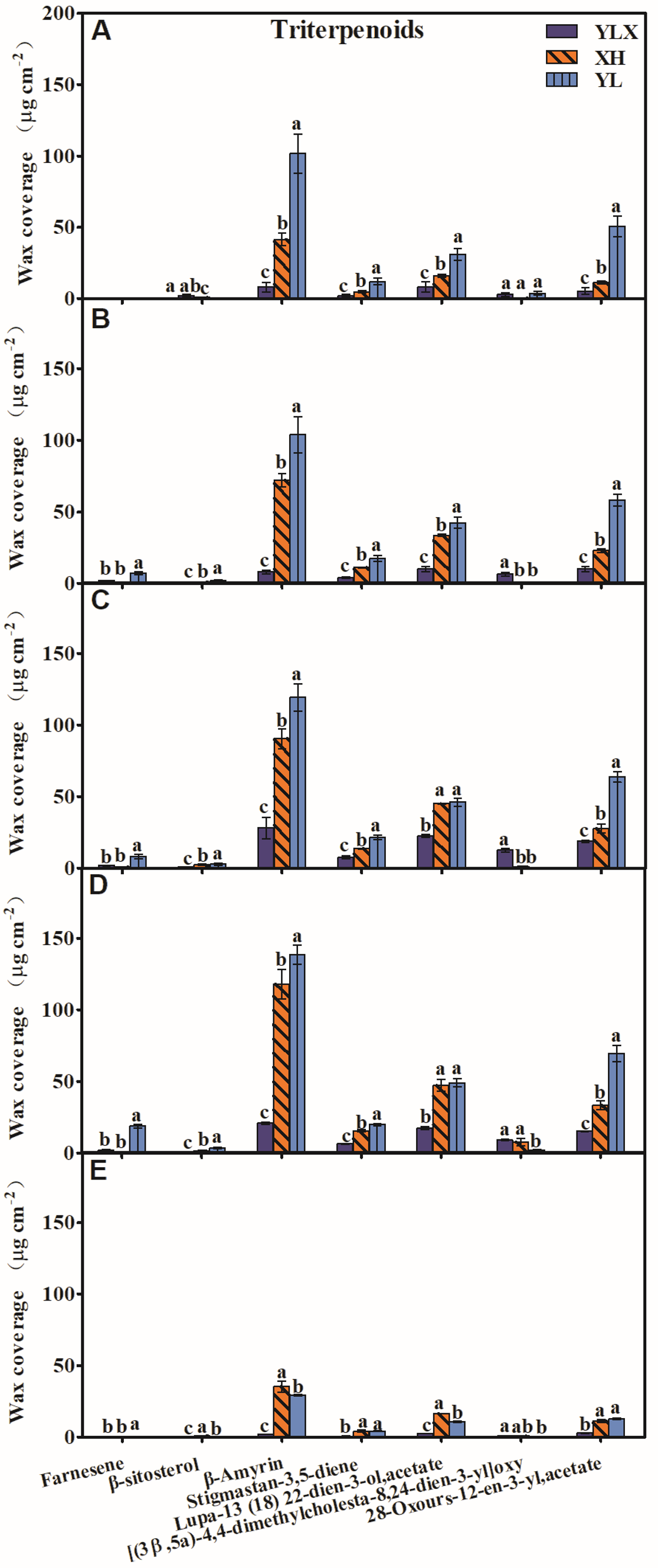

Supplement: Supplemental Information 7 — Data were mean ± SE (n = 3); different letters marked on the three columns of the same group indicate significant differences in data according to one-way ANOVA at p < 0.05. (A) day 0 (B) day 45 (C) day 90 (D) day 180 (E) day 270. Yuluxiang, Xuehua and Yali are abbreviated to YLX, XH and YL. [file peerj-10-14328-s007.png]

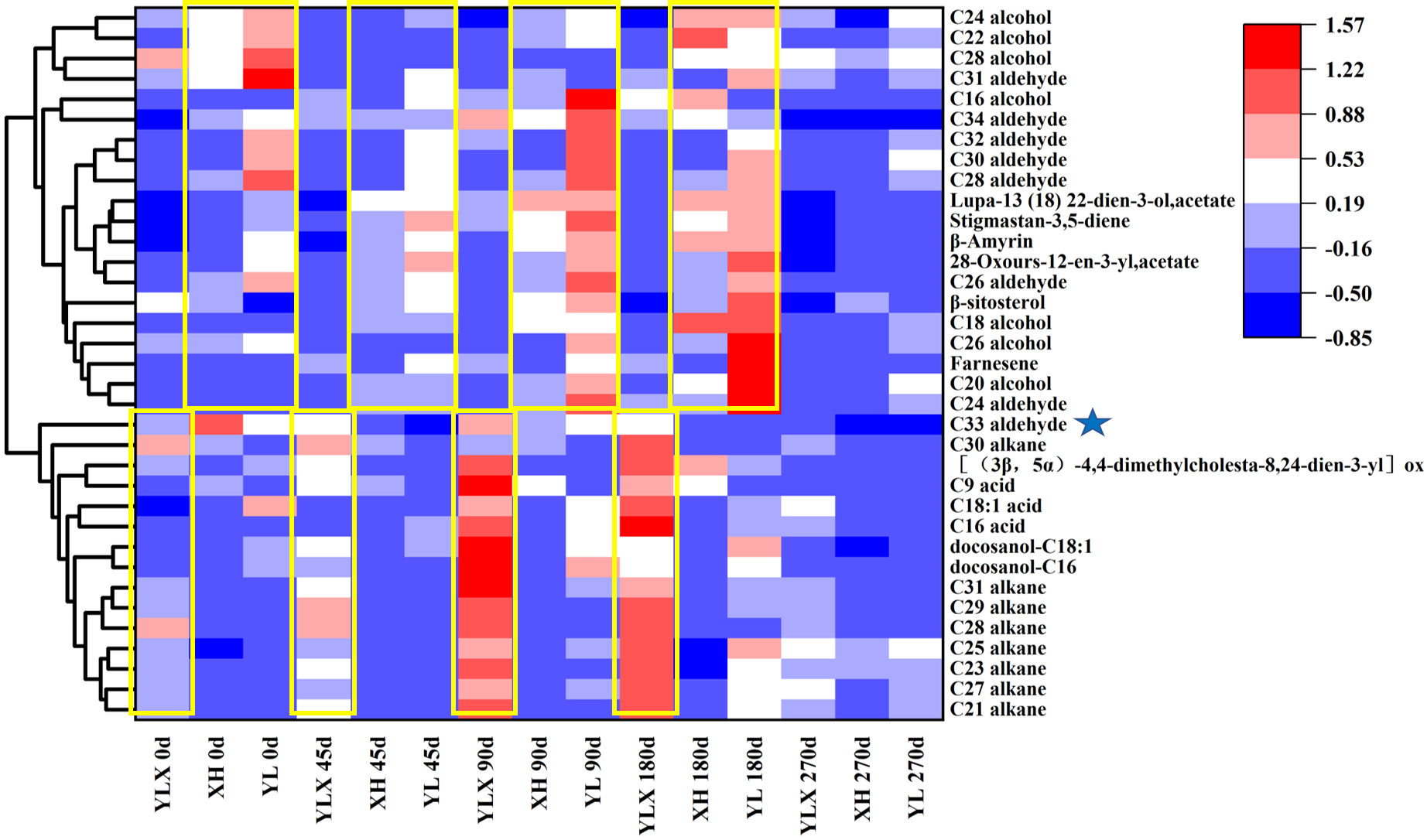

Supplement: Supplemental Information 8 — Color indicated wax composition contents, and each column represented a combination of variety and storage period. Yellow square areas represent varieties and time combinations with higher wax content after clustering. The wax component indicated by blue asterisk represents the dividing point of component clustering. [file peerj-10-14328-s008.png]
