# Supplementary material for: Changing surface wax compositions and related gene expression in three cultivars of Chinese pear fruits during cold storage
Source: PeerJ. 2022 Nov 1;10:e14328. doi: 10.7717/peerj.14328 (PMC9635359; doi:10.7717/peerj.14328)
Supplement: Supplemental Information 9 [file peerj-10-14328-s009.docx]

| Gene | Accession No. | Forward primer (5′-3′) | Reverse primer (5′-3′) |
| --- | --- | --- | --- |
| *LACS1* | XM_009364839.1 | AGAAGGCGAGGATGGCAA | CCAAGCATTCGATTTCCAGG |
| *LACS2* | XM_009347511.2 | CGGCGTATCGGAGCATTT | ATCGCAGCATCATAAACCTCTT |
| *KCS1L* | XM_009353114.2 | ATGGATGCTGCTTCTGTTCA | CTTGAGCTTGACGGACTGTAAG |
| *FDH* | XM_009360773.2 | TTCTCAGTGAGGGTGCAAAGG | AGCACTGAACACCAGCACAAG |
| *KCS2* | XM_009350716.1 | TCAAGGATGACGCTCTACAGGT | AACGCAATCTGCCACATCC |
| *KCS6* | XM_018647444.1 | GTATGTCAAGCTCGGCTACCAG | GACGAGGTCGAAGTGGAGTG |
| *KCS20* | XM_018646010.1 | TCTCGGTCACTTTGTGCTCC | GAAAAGCCAAGTTCTCGTCGTA |
| *GL8* | XM_009360989.2 | TCAACTGATGGTTATGCCCG | GCCATGCGTCAACAACAGA |
| *CER10* | XM_018644500.1 | AAGCCCAGATGGAAATGGAG | GATGGAAGCGGCAACTATGA |
| *CER60* | XM_021976238.1 | GGTCCTTGGGAAGACTGCAT | TGCGAGAATCGAGCAGAGAA |
| *LTPG1* | XM_009374000.2 | CGGTGAAGGGGATAAGGGAG | AATCGGAGACAGTGGCGTTT |
| *LTP3* | XM_009348637.1 | AGCTCCAACCTTGCACCATG | CAGGAAGCGATTCGGCATT |
| *LTP4* | XM_009373738.2 | TTTGGTGGTGGCCTTGAGC | TCCTGGCGATGCCGTTA |
| *ABCG11* | XM_009346319.2 | ATCAATGGGCATAAACAAG | CAGAAGCAGGACCAAAATA |
| *ABCG12* | XM_009376649.2 | GATCTAAGTGCGGTGCTTCC | GCCATGATCCTACCAGGCT |
| *CER1L* | XM_009338987.2 | TAACCACACCCGAGTCCATC | CATCATAGACCACAATGTCACAGG |
| *CAC3* | XM_009371940.2 | CCATTGGCTGTGCTAATACATTAC | GCTTTTCAGCAGCCTTTGG |
| *CAC3L* | XM_009360561.2 | TGAGAGCCCTAGGGAAGGTAA | CACTCCACCTTTGACATTGG |
| *DGAT1L* | XM_009363362.2 | TCGACTCGAACGTGAACCAG | TCTTGAACAGCATTGACGGACT |
| *WSD1L* | XM_009380488.2 | CTGGCTGATATGATGGCTAAGAAGT | CGAGATTGGCAGTCAGGTATGTG |
| *ACT2* | GU830959 | GGACATTCAACCCCTCGTCT | ATCCTTCTGACCCATACCAACC |
